# Delphinidin-Rich Maqui Berry Extract (Delphinol®) Lowers Fasting and Postprandial Glycemia and Insulinemia in Prediabetic Individuals during Oral Glucose Tolerance Tests

**DOI:** 10.1155/2016/9070537

**Published:** 2016-11-29

**Authors:** Jorge L. Alvarado, Andrés Leschot, Álvaro Olivera-Nappa, Ana-María Salgado, Hernán Rioseco, Carolina Lyon, Pilar Vigil

**Affiliations:** ^1^Reproductive Health Research Institute, Pontificia Universidad Católica de Chile, Santiago, Chile; ^2^MNL-Chile, Santiago, Chile; ^3^Centre for Biotechnology and Bioengineering, Department of Chemical Engineering and Biotechnology, University of Chile, Santiago, Chile

## Abstract

Delphinidin anthocyanins have previously been associated with the inhibition of glucose absorption. Blood glucose lowering effects have been ascribed to maqui berry (*Aristotelia chilensis*) extracts in humans after boiled rice consumption. In this study, we aimed to explore whether a standardized delphinidin-rich extract from maqui berry (Delphinol) affects glucose metabolism in prediabetic humans based on glycemia and insulinemia curves obtained from an oral glucose tolerance test (OGTT) after a challenge with pure glucose. Volunteers underwent four consecutive OGTTs with at least one week washout period, in which different doses of Delphinol were administered one hour before glucose intake. Delphinol significantly and dose-dependently lowered basal glycemia and insulinemia. Lower doses delayed postprandial glycemic and insulinemic peaks, while higher doses reversed this tendency. Glycemia peaks were dose-dependently lowered, while insulinemia peaks were higher for the lowest dose and lower for other doses. The total glucose available in blood was unaffected by treatments, while the total insulin availability was increased by low doses and decreased by the highest dose. Taken together, these open exploratory results suggest that Delphinol could be acting through three possible mechanisms: by inhibition of intestinal glucose transporters, by an incretin-mediated effect, or by improving insulin sensitivity.

## 1. Introduction

Frequent excessive postprandial glucose and insulin excursions represent a risk factor for developing diabetes, associated with impaired glucose tolerance (IGT) and impaired insulin tolerance (IIT), inflammation, dyslipidemia, *β*-cell dysfunction, and endothelial dysfunction [1]. The maintenance of healthy blood sugar levels and controlled carbohydrate metabolism is a rapidly growing concern in most developed countries and increasingly also in developing countries, due to the increased awareness of the hyperglycemia risks resulting from unhealthy diets and sedentary lifestyle [2]. Further to dietary self-limitation and physical activity efforts, consumption of plant secondary metabolites may substantially contribute to improving carbohydrate and lipid metabolism [3–6].

Long term epidemiologic studies have pointed to dietary factors affecting the risk for developing diabetes. Investigation of data from the Nurses Health Studies (NHS) has resulted in interesting findings related to elevated regular consumption of different flavonoid classes and disease risk reduction [7]. Higher consumption of anthocyanins was associated with lower risk for type II diabetes in US adults, based on the follow-up of 70359 women in the NHS (1984–2008) and 89201 women in NHSII (1991–2007) and also 41334 men in the Health Professionals Follow-Up Study (1986–2006) [7]. Interestingly, this study found no significant correlation between other flavonoid subclasses and even total flavonoid consumption related to risk reduction for type II diabetes. A follow-up of the NHS II (93600 women) suggested that anthocyanin intake in the form of blueberries and strawberries would correlate with decreased myocardial infarction risk [8]. A recent epidemiologic study suggests that regular higher intake of flavonoid species anthocyanins, flavones, and flavanones is associated with greater likelihood for good health and wellbeing in individuals surviving to older ages [9].

Polyphenols are well described to exhibit inhibitory effects on *α*-glucosidase and *α*-amylase enzyme activities, thus delaying absorption of complex food carbohydrates [10]. Particularly, the oligomeric proanthocyanidins potently delay hydrolysis of starchy foods to glucose, some of which appear to be more effective than acarbose medication [11]. Consumption of anthocyanin-rich crowberry-fortified blackcurrant juice was described to attenuate significantly the postprandial blood glucose and insulin peak 90 minutes after glucose challenge, as compared to consumption of the same sugared beverage void of crowberry fortification [12].

Delphinidin anthocyanins extracted from maqui berries (*Aristotelia chilensis*), indigenous to Chile, are especially rich in glucoside and sambubioside derivatives. They have recently been ascribed to inhibit the sodium-glucose cotransporter type 1 (SGLT1) in rat duodenum. Delphinol, a proprietary maqui berry extract with a standardized content of 25% w/w delphinidin glycosides and 35% total anthocyanins, was found to significantly inhibit postprandial blood glucose 60 and 90 minutes after boiled rice intake [13], with a single 200 mg dose before food consumption.

We here describe the results of open exploratory investigations on the effect of different doses of Delphinol on blood glucose and insulin in fasting conditions and postprandial effects after glucose ingestion, by applying a standard oral glucose tolerance test (OGTT) in study participants with impaired glucose tolerance.

## 2. Methods

### 2.1. Study Population

Potential subjects presenting with either a family history of type 2 diabetes, hypertension, body mass index greater than 23 kg/m^2^, or dyslipidemia, aged between 18 and 50 years of both genders, were invited to participate in a screening procedure to identify suitability to meet inclusion criteria.

Inclusion criteria comprised the following: (1) age between 18 and 50 years; (2) abnormal response to OGTT, as the result of altered blood glucose values (basal glucose ≥ 100 mg/dL, any intermediate glucose value at 30, 60, or 90 minutes ≥ 160 mg/dL, or glucose at 120 minutes ≥ 140 mg/dL) or blood insulin values (basal insulin ≥ 15 *μ*IU/mL, any intermediate insulin value at 30, 60, or 90 minutes ≥ 100 *μ*IU/mL, or insulin at 120 minutes ≥ 60 *μ*IU/mL).

Exclusion criteria comprised fasting blood glucose levels ≥ 180 mg/dL, pregnancy, hormonal therapy with sexual steroids, cardiovascular disease requiring medication, hypoglycemic medication use, allergies, and the inability to follow instructions.

Prior to enrolment, all participants were introduced in detail to the purpose and rationale of the study and the product they would be taking as well as the investigational procedures they would be exposed to. All participants provided their written informed consent for participation in the research project. The present study was conducted according to the Declaration of Helsinki guidelines. The study protocol was approved by the ethical committee of Mutual de Seguridad, Santiago, Chile. Subjects were permitted to discontinue participation at any time without providing reasons. Pregnancy testing in women with childbearing potential was only performed at the first visit. During the first health checkup, specimens were collected from all enrolled participants for blood rheology, standard blood chemistry, lipid profiling, and complete urine analysis.

### 2.2. Study Design and Protocol

This investigation was an open exploratory study initiated for identifying acute dose effects of a standardized maqui berry extract (Delphinol) on postprandial blood glucose. Delphinol is mainly composed of polyphenols and is standardized to contain more than 25% delphinidin glycosides by HPLC area under the curve (AUC), using purified reference standards delphinidin-3-sambubioside-5-glucoside, delphinidin-3,5-diglucoside, delphinidin-3-sambubioside, and delphinidin-3-glucoside. Furthermore, Delphinol is standardized to contain no less than 35% total anthocyanins, comprising also cyanidin glycosides with the same glycosylation patterns as those in delphinidins. Many further nonanthocyanin flavonoid species with the same glycosylation patterns are present in Delphinol [14, unpublished results]. Delphinol has been shown to be safe in acute and chronic toxicity assessments at doses of 1 g/kg body weight. Delphinol is commercialized as a dietary supplement in most parts of the developed world, predominantly as an antioxidant [15], and for blood glucose management [16]. Capsules bearing 60, 120, and 180 mg Delphinol (batch 13156, MNL, Santiago, Chile), manufactured by Barnafi Krause Farmacéutica SA (Santiago, Chile), were used in this study.

### 2.3. Subjects

According to the inclusion criteria, a total number of 36 prediabetic subjects were initially enrolled, 20 women and 16 men aged 19 to 50 years. Additional seven participants were recruited at a later time to increase the statistical power. This group comprised 4 women and 3 men aged 37 to 43 years, all of them being prediabetic. In the first visit, the health status of the subjects was checked by an interview to register their clinical history and a complete physical examination. Routine analyses (complete blood count, biochemical and lipid profiles) and a diagnostic OGTT were also performed. At the end of each visit, participating volunteers were interviewed for adverse effects.

### 2.4. Oral Glucose Tolerance Test and Insulin Measurements

Study participants were screened for eligibility by means of an oral glucose tolerance test after Trutol® (75 g of glucose as a 296 mL aqueous solution, Thermoscientific, Pittsburgh, PA, USA) intake, measuring basal (fasting), 30-, 60-, 90-, and 120-minute blood glucose and insulin levels, with glycemia and/or insulinemia exceeding values as in the aforementioned inclusion criteria.

**Figure 1:**
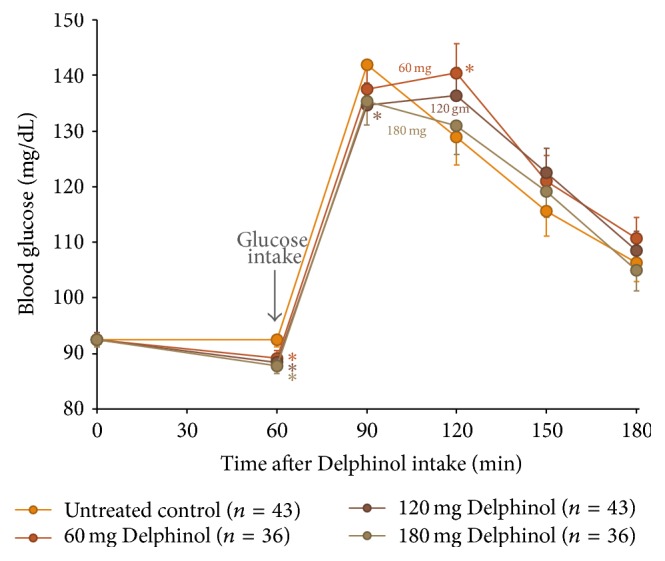
Mean glycemia variation during OGTT for all volunteers treated with four different Delphinol doses of nil (control), 60, 120, and 180 mg, at four different occasions with several washout days in between experiments. Sixty minutes after Delphinol intake, participants presented with dose-dependent lowering of basal blood glucose. At this time, 75 g of glucose was consumed and resulting postprandial glucose levels are presented. Statistically significant (*α* = 0.05) altered values compared to untreated control are indicated by an asterisk.

For the study of dose effects, each participating subject was given a single dose of Delphinol (nil, 60, 120, or 180 mg) on different test days, with minimum of one-week intermission between tests. After an overnight (10–12 hours) fasting period, subjects were cannulated after arrival at the medical facility and remained lying on a hospital bed over the entire investigational period. An initial 4 mL blood sample was taken and the corresponding dose of Delphinol was subsequently administered. One hour later, participants were subjected to an OGTT, swiftly ingesting a standard 75 g glucose solution (Trutol). For the OGTT, in total, five 4 mL blood samples were taken in intervals of 30 min. The first blood sample was collected before glucose ingestion, as illustrated in Figure 1. The subsequent four samples were collected in 30-minute intervals during two hours after glucose intake. All samples were collected in Becton Dickinson (BD) Vacutainer tubes containing EDTA and sodium fluoride, centrifuged at 4.000 rpm on an ALC PK 120 centrifuge for 5 minutes, and stored at 4°C for laboratory measurements.

Glucose was estimated by a GOD-PAP colorimetric assay using a Selectra autoanalyzer (Vitalab, Smithfield, USA). Calibration was performed daily according to lab protocols. All samples were processed following standard procedures. Insulin was analyzed by direct chemiluminescence using an ADVIA Centaur XP Immunoassay System (Siemens, Erlangen, Germany).

### 2.5. Statistical Analyses

Statistical analyses were carried out using MINITAB 17 (Minitab Inc., State College, PA, USA), Origin Pro 8 (OriginLab Corp., Northampton, MA, USA), and SAS 9 (SAS Institute, Cary, NC, USA). One-tailed paired comparisons were used to identify significant differences in basal glucose values at a 5% significance level (*α* = 0.05) after ingestion of different Delphinol doses. For the comparison of different doses on subjects, repeated measures mixed ANOVA model was used in which subjects were treated as a random effect and doses as a repeated effect. The variance-covariance matrix was modeled using the options for the repeated statement provided in Proc MIXED (SAS) with a Toeplitz structure to allow for an autoregressive covariance model. After the general repeated measures ANOVA, least-squares-means multiple comparisons were performed among doses. For the multiple comparisons, a Dunnett-HSU approach was applied using Proc MIXED (SAS Institute). One-tailed hypothesis testing was used to compare the control dose (nil) to 60, 120, and 180 mg doses. All reported mean values are least-square means due to the nonbalanced mixed model fitting the overall comparisons model.

Due to practical reasons and design limitations, after recruitment of seven additional subjects,* a priori* (planned) comparisons between control dose (nil) and the 120 mg dose were done using one-tailed paired *t*-tests for glucose and insulin. For this comparison, the complete dataset was analyzed using the same statistical model structure but comparisons were limited only to the control and 120 mg dose.

## 3. Results

### 3.1. Study Population

A total number of 36 prediabetic subjects (20 women and 16 men) were initially enrolled. The mean age was 30.1 (SD = 9.64; range = 20–50) years for women and 32.6 (SD = 8.92; range = 19–49) years for men, BMI was 29.57 (SD = 5.19; range = 20.1–39.2) kg/m^2^ for women and 31.97 (SD = 24.3; range = 24.3–48.5) kg/m^2^ for men, and fasting plasma glucose at enrolment was 88.4 (SD = 10.82; range = 74.0–117.0) mg/dL for women and 95.75 (SD = 7.05; range = 83.0–110.0) mg/dL for men. Specifically, for* a priori* statistical evaluations at 120 mg Delphinol, additional seven participants were recruited (4 women and 3 men). For those patients, the mean average age was 32.5 (SD = 11.12; range 37–43) years for women and 32.0 (SD = 14.73; range 39–43) years for men, BMI was 24.63 (SD = 1.46; range = 23.7–26.8) kg/m^2^ for women and 24.93 (SD = 2.97; range 22.2–28.1 kg/m^2^) for men, and fasting plasma glucose at enrolment was 91.0 (SD = 15.74; range = 75–109) mg/dL for women and 99.0 (SD = 6.55; range = 93–106) mg/dL for men.

### 3.2. Dose Effects of Delphinol on Fasting Glucose and Insulin

As detailed in Table 1, the mean overnight fasting glucose and insulin level of subjects, investigated on four different occasions, decreased within 60 minutes after a single intake of Delphinol in a dose-dependent manner. The decrease was statistically significant for all doses as compared to the non-Delphinol-treated control.

The decrease of fasting blood glucose subsequent to an acute intake of Delphinol coincided with a dose-dependent and significant decrease of fasting insulin as compared to the untreated control. One-tailed paired comparisons showed highly significant differences between basal glucose mean values and after ingestion of all three doses of Delphinol (Table 1) at a 5% significance level (*α* = 0.05). Regarding estimated effect sizes, mean reductions of 2.7, 3.14, and 3.61 (mg/dL) were observed for the 60, 120, and 180 mg dose, respectively.

One-tailed paired comparisons showed highly significant differences between basal insulin mean values and after ingestion of 180 mg of Delphinol (Table 1) at a 5% significance level (*α* = 0.05). Regarding estimated effect size, a mean reduction of 3.4 (*μ*IU/mL) was observed for the 180 mg dose.

### 3.3. Dose Effects of Delphinol on Postprandial Glucose in an OGTT

All subjects tolerated the treatment with Delphinol well, with no adverse reactions reported during interviews of participants. None of the participants departed from the study prior to completion of all experiments.

**Figure 2:**
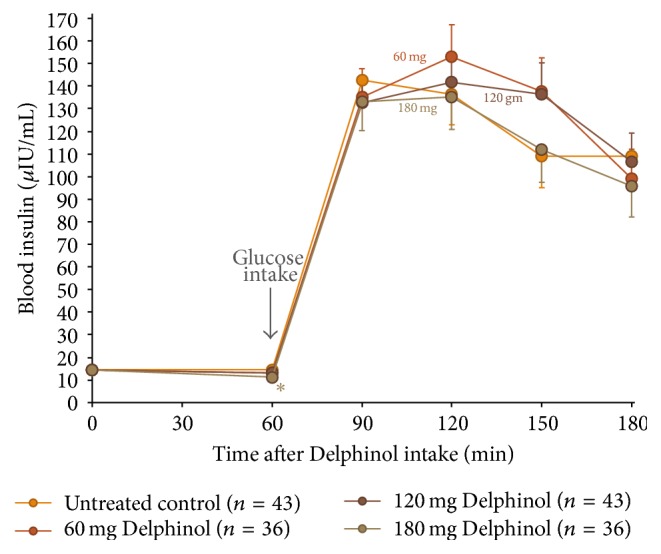
Mean insulinemia variation during OGTT for all volunteers treated with four different Delphinol doses of nil (control), 60, 120, and 180 mg, at four different occasions with several washout days in between experiments. Sixty minutes after Delphinol intake, participants presented with dose-dependent lowering of basal blood glucose. At this time, 75 g of glucose was consumed and resulting postprandial glucose levels are presented. Statistically significant (*α* = 0.05) altered values compared to untreated control are indicated by an asterisk.

**Figure 3:**
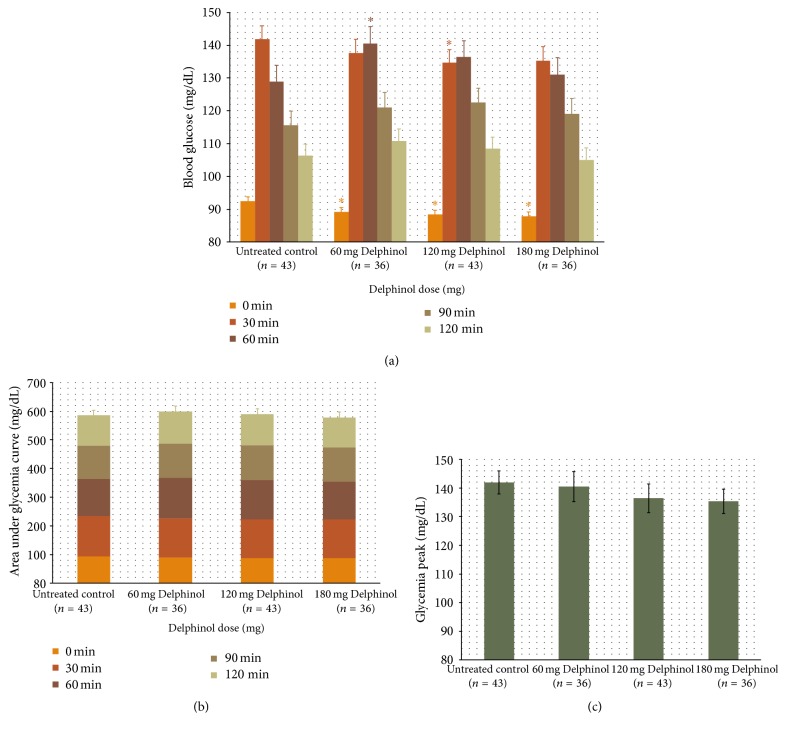
(a) Mean glycemia variation during OGTT for all volunteers treated with each separate Delphinol dose. Basal and postprandial glycemia levels are presented, in order to compare the tendencies observed at each time point depending on the dose administered. (b) Time normalized area under the glycemia curve for each dose with the contribution of each time segment. (c) Maximum glycemia peak for each dose, with regression line and 95% confidence intervals, slope value, and associated significance level. Statistically significant (*α* = 0.05) altered values compared to untreated control are indicated by an asterisk.

**Figure 4:**
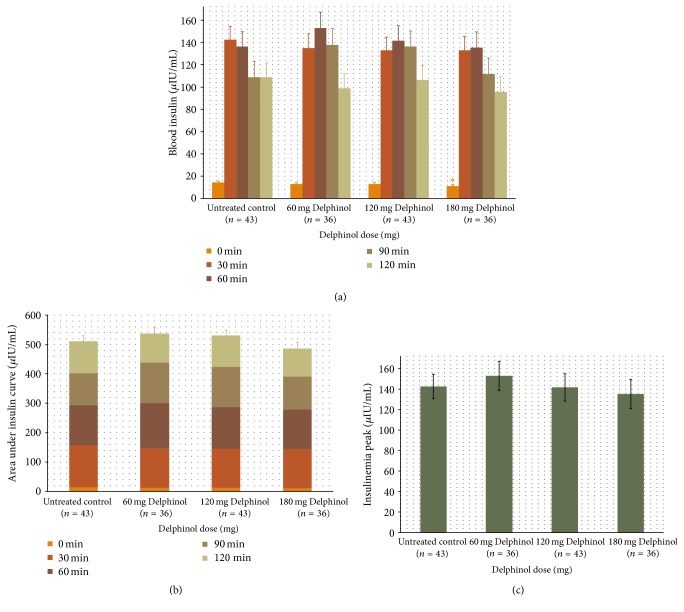
(a) Mean insulinemia variation during OGTT for all volunteers treated with each separate Delphinol dose. Basal and postprandial insulinemia levels are presented, in order to compare the tendencies observed at each time point depending on the dose administered. (b) Time normalized area under the insulinemia curve for each dose with the contribution of each time segment. (c) Maximum insulinemia peak for each dose. Statistically significant (*α* = 0.05) altered values compared to untreated control are indicated by an asterisk.

As presented in Figures 1 and 2, blood glucose and insulin OGTT curves present distinctly different kinetics in response to the Delphinol dose applied. Within thirty minutes after glucose ingestion, a dose-dependent effect of Delphinol on the rapidly increasing postprandial glucose was observed. For untreated subjects, blood glucose rose markedly higher during the diagnostic tests in absence of Delphinol than with prior intake of Delphinol (Figure 3(c)). A linear regression for the glucose maximum peak height as a function of the applied Delphinol dose calculated a significant nonzero negative slope (*p* = 0.0273). Insulinemia reached higher peak values in subjects treated with Delphinol for the lowest 60 mg dose, while for higher doses the tendency was to equate the maximum values of the control, with a slight tendency to even lower values for the 180 mg dose (Figure 4(c)).

Borderline statistical significance versus untreated control was identified in glycemia for 120 mg (*p* = 0.117) and 180 mg (*p* = 0.126) Delphinol 30 minutes after glucose intake. Based on these results, we chose to increase the sample size for the 120 mg dose in order to corroborate these results and elevate the statistical power of the test. Seven additional prediabetic subjects, 4 women and 3 men, aged 37 to 43 years, were recruited and investigated by the same procedures as described earlier. The glucose and insulin single* a priori* comparisons, including the 7 additional recruits, testing 120 mg Delphinol versus control, are presented in Table 2. As a result, from the a priori comparisons (Table 2), we found that application of a single 120 mg Delphinol dose significantly decreased 30 min postprandial (OGTT) blood glucose (*p* < 0.05), whereas the corresponding postprandial lowering of insulin remained statistically insignificant.

During the subsequent period from 30 min to 60 min after glucose intake, blood glucose and insulin curves show surprisingly diverging developments in response to the applied Delphinol dose. As shown in Figure 3(a), in Delphinol-treated subjects, glycemia peaks showed a dose-dependent retardation of the maximum value for the 60 and 120 mg doses, compared to the untreated subjects, and a continued rise of glucose and insulin values persists until 60 min after glucose intake. For the highest Delphinol dose of 180 mg, this peak delay is reversed back to 30 minutes after glucose challenge and a more pronounced decrease is attained in the maximum glycemia value than that for lower doses. As seen in Figure 4(a), insulinemia also showed a similar delay in the appearance of peak maxima, but the reversal of this tendency for the 180 mg dose was less conspicuous than that in the glycemia curve. Nevertheless, insulinemia values 120 minutes after the glucose challenge were smaller than the control with all doses and particularly with the highest dose.

Surprisingly, the lowest applied dose of 60 mg Delphinol presents with the highest glucose level at 60 min, reaching borderline statistical significance (*p* = 0.056) versus untreated control (Figure 3(a)). The insulin level recorded for the 60 mg dose correspondingly presents with the highest value of all four applied Delphinol doses at this time point (Figure 4(a)). It is noteworthy that 60 min after glucose challenge the lowest glucose level was found for the untreated control, whereas the lowest insulin value was presented with 180 mg Delphinol.

A noticeable divergence related to dose effects is apparent for the insulin levels 90 min after glucose challenge. Whereas higher glucose values at this time with 60 and 120 mg Delphinol are reflected by correspondingly high insulin values, the values decline in the untreated control and likewise, to the same extent, with the 180 mg dose. Two hours after glucose intake, glucose levels reach the lowest values for the untreated control and 180 mg Delphinol dose. Remarkably, with 180 mg Delphinol, the insulin level remained at the lowest levels throughout all investigational time points.

In order to estimate the total amount of glucose and insulin available in the blood during the OGTT, we calculated a time normalized area under the curve (AUC) for glycemia and insulinemia values. For glycemia, this value is equivalent to the glycemic index (GI) [1]. Figures 3(b) and 4(b) show these results. No significant difference was found for any Delphinol dose compared to the control, but tendencies can be observed for both curves. Glucose availability tended to be almost constant for all groups. Insulin showed a clearer trend, with more availability for the lowest 60 mg dose and a discernible tendency to decrease as dose increases. In fact, for the 120 mg dose, the availability is similar to that of the control, whereas for the highest 180 mg dose this value is inferior to that of the control. Interestingly, the tendencies of insulin availability at different doses parallel those of maximum insulin peak heights.

## 4. Discussion

Dietary polyphenols are well described to contribute to healthier blood glucose values, based predominantly on inhibition of *α*-glucosidase and *α*-amylase activities [8]. While oligomeric proanthocyanidins potently inhibit *α*-glucosidase activity and effectively lower postprandial blood glucose following starchy meals, the absorption of monosaccharides, especially glucose itself, remains little affected by most flavonoid species. Berries of various natures, consumed as pulp or juice, have repeatedly been demonstrated to limit the rise of blood glucose and insulin in response to sucrose challenge. In all these studies, inhibition of *α*-glucosidase was ascribed to delay hydrolysis of sucrose to glucose and fructose [10, 12].

Some polyphenolic species such as epicatechin gallate have been ascribed to show inhibitory effects to sodium-glucose cotransporter activity [3]. Delphinidins have been demonstrated to inhibit SGLT1 activity in rat duodenum in Ussing chambers, and acute intake of 200 mg Delphinol correspondingly significantly lowered postprandial blood glucose and insulin following a 75 g boiled rice challenge in a healthy human pilot trial [13]. However, clinical evidence for inhibition of glucose absorption, such as in an oral glucose tolerance test, to date remains absent for delphinidin aglycon as well as for delphinidin-derived anthocyanins. Hence, we conducted a study using a naturally delphinidin-rich maqui berry extract (Delphinol) in order to investigate dose related inhibition of glucose absorption in human volunteers using standard OGTT. We chose to study prediabetic subjects in expectation of greater effects on postprandial glucose alterations following acute intake of Delphinol. Furthermore, no previous study has clinically established the Delphinol minimum acute dose required for significant lowering of postprandial blood glucose in OGTT. This prompted us to test acute doses of 60, 120, and 180 mg Delphinol versus untreated control in an OGTT in repeated investigations with the same subjects.

Unexpectedly, we discovered that acute intake of Delphinol alone, in the absence of any carbohydrate exposure, at all Delphinol doses tested, simultaneously lowered both postprandial fasting blood glucose and insulin one hour after intake, in a dose-dependent and significant fashion. An acute simultaneous reduction of blood glucose and insulin with dietary polyphenols at fasting conditions, to our knowledge, has not been described before. The investigation of underlying mechanisms of action related to this observation was beyond the scope of the research project. Since Delphinol was previously described to inhibit SGLT1 activity in small intestine, it was intriguing to speculate whether Delphinol may potentially inhibit SGLT1 or SGLT2 in kidneys, affecting renal glucose reabsorption with glucose lost to urine [17]. Investigation of possible glucosuria was not part of the research protocol; however, a* post hoc* investigation of only three subject volunteers participating in the trial showed that, following a single intake of 180 mg Delphinol at fasting conditions, urine collected over the subsequent hour did not contain measurable quantities of glucose (data not shown). Though the observation on the absence of glucosuria is limited, this finding nonetheless suggests that delphinidin glycosides in maqui berries may not lower fasting glucose as a result of glucosuria.

The effects of Delphinol presented here shall be of particular interest for individuals with glucose intolerance and insulin resistance, as pancreatic *β*-cells may be less burdened by excess insulin secretion. The insulin sparing effect identified for Delphinol is particularly appreciable as it manifests also at fasting conditions. The observation that insulin levels were lowered following Delphinol ingestion (prior to OGTT), and with a single dose of 180 mg persisted lower throughout the monitored 120 min period, as compared to untreated control as well as lower Delphinol doses, points to potential pancreatic health contributions. This discovery of Delphinol effects on insulin merits future research, which may explore fasting glucose and pancreas function related to insulin response improvement, especially following regular supplementation with Delphinol. Although sugar metabolism effects of other polyphenols present in maqui berry may not be excluded at this point, it has already been described that a particular anthocyanin, delphinidin 3-sambubioside 5-glucoside (D3S5G), displays insulin-like effects in muscle and liver cells and seems to be partially responsible for the antidiabetic effect of maqui berry extracts [18].

We found that applied Delphinol doses significantly lowered blood glucose 30 minutes after glucose challenge, which may arguably be partially attributed to the drop in fasting glucose subsequent to Delphinol intake. Yet, our findings confirm that Delphinol decreases absorption of dietary glucose further to previous investigations of Törrönen and coworkers who gave starchy foods to subjects [12]. Our results are coherent with the SGLT1 inhibition by delphinidins described by this study.

It is noteworthy that the postprandial blood glucose and insulin dynamics are profoundly altered in relation to the Delphinol dose applied, from the time point 30 min after glucose ingestion. Surprisingly, the 60 min postprandial blood glucose values are present in inverse order with respect to the Delphinol dose applied to subjects. Whereas in untreated control blood glucose dropped to an average of 126.8 mg/mL 60 min after glucose challenge, the average values with Delphinol ranged higher at 129.1, 134.1, and 139.1 mg/mL, for 180, 120, and 60 mg Delphinol, respectively. This observation may appear to be contradictory to inhibition of SGLT1 with Delphinol. However, it is noteworthy that supplementation with Delphinol occurred one hour prior to glucose challenge and delphinidin glycosides may potentially no longer persist at physiologically fully active concentrations in the small intestine two hours past Delphinol intake.

In the same line, the observed delay of blood glucose peaks can be seen as the effect of a partial SGLT1 inhibition at lower dosages. This inhibition is expected to be higher in the proximal segments of the intestine, which would imply a slower glucose absorption in the duodenum and a retarded absorption in the following intestinal segments. As doses grow higher, inhibition is expected to be more complete, including inhibition at the same higher level in both proximal and more distal intestinal segments, which would cause a recovery of the form of the glucose curve and a concomitant lowering of blood glucose, as observed with the 180 mg Delphinol dose. Interestingly, the peak retarding effect induced by Delphinol in low doses partly mimics the shape of curves of lower glycemic index foods [1].

However, none of the above-discussed mechanisms could account for the rise of the insulin peak and the higher insulin availability observed with the lowest 60 mg Delphinol dose (Figures 2 and 4). In fact, while the tendency for glucose peaks is to decrease as the Delphinol dose increases (Figures 1 and 3) and total glucose availability remains constant during the OGTT for all treatment groups, this better control seems to require higher absolute levels of insulin and higher insulin secretion for the lowest dose tested. This is counterintuitive, since lower blood glucose cannot by itself trigger more insulin secretion in normal conditions. Therefore, apart from the possible inhibition of intestinal glucose transporters, Delphinol should have a more direct effect on insulin secretion. Whether this effect is mediated by incretins or is a direct action on pancreatic *β*-cells cannot be ruled out by the current evidence.

Strikingly, the case of the highest 180 mg dose presents a radically different effect: its glycemia peak is the lowest among all treatments and control, and the total amount of glucose in blood during the OGTT remains the same, while the insulin peak is also the smallest. However, the total amount of circulating insulin required for this is also the lowest among all treatments and control. This evidence indicates that the same amount of glucose is managed more efficiently with less insulin, which in turn suggests that Delphinol could also improve insulin sensitivity of target tissues in a direct or indirect manner, making this a third possible mechanism by which Delphinol could act on glucose metabolism.

Other groups investigating polyphenol impact on postprandial glucose applied anthocyanins as blackcurrant juice, fortified with additional crowberry (*Empetrum nigrum*) powder (100 g/L) and adding sucrose (50 g/L) to the juice [12]. Plasma glucose and insulin were lowered with crowberry-fortified sugared blackcurrant juice 30 min past consumption, though nonsignificant compared to the sweetened blackcurrant juice alone. Interestingly, significantly higher blood glucose and insulin levels were identified 90 min after consumption of sucrose-sweetened and crowberry-fortified blackcurrant juice, as compared to sweetened blackcurrant juice without crowberry. These postprandial glucose and insulin kinetics are in conformance with our findings, showing elevated glucose and insulin at 60 and 90 min, resulting from anthocyanin consumption. Törrönen and coworkers attribute the attenuation of postprandial glucose and insulin to *α*-glucosidase inhibition, resulting in delayed sucrose hydrolysis, and speculate on the possibility for additionally impaired intestinal glucose transport [12]. As we used glucose in our experimental setting, any possible effects of Delphinol on carbohydrate hydrolysis are ruled out.

Hoggard et al. measured postprandial OGTT curves after Polycal (49% polysaccharides, 12% glucose, 0.6% maltose) consumption, on 8 patients with diabetes or IGT to which 470 mg of bilberry (*Vaccinium myrtillus*) extract was given before the trial [19]. In this study, total anthocyanins doses were much higher than in our trial. Since they used a mixture of glucose and polysaccharides on patients with more advanced disease states than in our study, their results are not comparable to our findings. Indeed, they reported statistically significant lower postprandial glucose levels only 120, 150, and 180 minutes after Polycal consumption, in contrast to our findings, which suggests intestinal absorption played a more delayed role in their experimental setting.

## 5. Conclusions

We have demonstrated for the first time that a maqui berry extract standardized in its anthocyanin content (Delphinol) is able to simultaneously reduce fasting blood glucose and insulin levels in prediabetic patients when administered in a single dose. A significant effect was obtained even for very low doses of Delphinol: only 60 mg of Delphinol, corresponding to 21 mg of maqui berry anthocyanins, was statistically significant for the basal glucose drop. A dose of 180 mg of Delphinol, corresponding to 63 mg of maqui berry anthocyanins, was effective in significantly decreasing fasting insulin levels. These results are unique because these trials were performed in prediabetic humans and not in an animal model.

Furthermore, in contrast to previous studies that used disaccharides (sucrose) or complex polysaccharides sources (Polycal or boiled rice) to measure OGTT, we used pure glucose, the gold standard procedure. In these conditions, we demonstrated that a single 120 mg Delphinol dose decreases the glycemic peak 30 minutes after glucose challenge in a single dose trial. Our results show also tendencies for maximum postprandial blood glucose peaks to decrease with all tested Delphinol doses in a dose-dependent manner. Trends were also detected for insulin peaks, which tend to decrease with higher Delphinol doses. A similar decreasing tendency with higher Delphinol doses was observed for the total amount of insulin available in blood during the OGTT. Even though significant differences and trends were only demonstrated for some measures, clear nonlinear tendencies are observed for many parameters, but formal demonstration of statistical significance would require the development of new statistical tests for trends. In a simpler approach, for 30 min glycemia measures, we demonstrated that observed differences were easily resolved by including only ~20% more volunteers in the study, in order to reach statistical significance.

Although it is too soon to describe a potential biochemical mechanism of action by which Delphinol is able to decrease blood glucose and insulin levels, taken together, our results suggest that Delphinol could be acting on glucose metabolism by three different possible mechanisms: (1) by inhibition of intestinal glucose transporters, (2) by an incretin-mediated effect on insulin secretion, or (3) by improving the insulin sensitivity in target tissues. These possible mechanisms are in line with the results of Rojo et al. [18], which describe insulin-like effects in liver and muscle cells of delphinidin 3-sambubioside 5-glucoside, one of the main anthocyanins of maqui berry, and identify this particular purified anthocyanin as, at least partially, responsible of the antidiabetic effects. However, in the present work, we cannot rule out that other polyphenol components of Delphinol could also be synergetically having insulin-like effects, as described, for example, by Cameron et al. [20]. We can discard inhibition of *β*-glucosidases, in our particular case, as we used pure glucose as the OGTT challenge. The exact nature of these putative mechanisms should be the research matter of future studies on maqui berry extract, in attendance to the utility that they may have in the management of chronic glucose metabolism conditions, such as metabolic syndrome and prediabetes. In this sense, this work adds to previous works and is part of a continuous and ongoing effort to better characterize maqui berry extracts, find effective doses, measure effects on glucose metabolism, and shed light on possible mechanisms of action for standardized maqui berry extracts.

## Figures and Tables

**(a):** 

Delphinol dose (mg)	Basal glucose (mg/dL)	Dunnett HSU comparison		
	60 min after Delphinol intake			
	Mean value and adjusted SE			
		Dose (mg)	Difference of least squares means	Adjusted *P*

0	91.67; 1.37			
60	88.94; 1.37	0 versus 60	2.72	0.037
120	88.53; 1.37	0 versus 120	3.14	0.034
180	88.06; 1.37	0 versus 180	3.61	0.04

**(b):** 

Delphinol dose (mg)	Basal insulin (*μ*IU/mL)	Dunnett HSU comparison		
	60 min after Delphinol intake			
	Mean value and adjusted SE			
		Dose (mg)	Difference of least squares means	Adjusted *P*

0	15.46; 1.15			
60	13.78; 1.15	0 versus 60	1.68	0.072
120	13.79; 1.15	0 versus 120	1.66	0.079
180	12.05; 1.15	0 versus 180	3.4	<0.001

**(a):** 

Dose (mg)	Basal glucose (mg/dL)	Difference of least square means	30-minute glucose (mg/dL)	*p* value
0	92.45; 1.29		141.93; 4.03	
120	88.36; 1.29	4.08	134.63; 4.03	0.048

**(b):** 

Dose (mg)	Basal insulin (*μ*IU/mL)	Difference of least square means	30-minute insulin (*μ*IU/mL)	*p* value
0	14.57; 1.06		142.64; 11.90	
120	13.08; 1.06	1.49	132.84; 12.60	0.19

## Abbreviations

SGLT1: Sodium-glucose cotransporter type 1
SGLT2: Sodium-glucose cotransporter type 2
OGTT: Oral glucose tolerance test
IGT: Impaired glucose tolerance
IIT: Impaired insulin tolerance.
